# Brivaracetam Use in Managing Seizures Following Traumatic Brain Injury

**DOI:** 10.7759/cureus.101198

**Published:** 2026-01-09

**Authors:** Alisha Ali, Zanib Javed, Ahsan Ali Khan, Altaf Ali Laghari, Muhammad Ehsan Bari

**Affiliations:** 1 Neurosurgery, Aga Khan University Hospital, Karachi, PAK; 2 Surgery, Aga Khan University Hospital, Karachi, PAK

**Keywords:** brivaracetam, levetiracetam, neurobehavioral outcomes, post-traumatic seizures, seizure prophylaxis, traumatic brain injury

## Abstract

Background: Traumatic brain injury (TBI) is a major cause of disability and mortality worldwide, frequently complicated by post-traumatic seizures (PTS). Levetiracetam (LEV) is widely used for seizure prophylaxis but is often associated with behavioral adverse effects. Brivaracetam (BRV), a newer antiseizure medication with higher synaptic vesicle 2A (SV2A) affinity, may offer similar efficacy with improved tolerability. This study compared the effectiveness and neurobehavioral outcomes of BRV versus LEV in TBI patients.

Methods: This prospective cohort study was conducted in the Department of Neurosurgery at a tertiary care hospital. A total of 132 adults with neuroimaging-confirmed TBI were enrolled and followed for six months. Patients were allocated into two groups based on the antiseizure medication prescribed at the treating physician’s discretion: Group A received BRV and Group B received LEV. The primary outcome was the occurrence of post-traumatic seizures, classified as early (<7 days) or late (>7 days post-injury). Secondary outcomes included functional recovery assessed by the Glasgow Outcome Scale-Extended (GOSE) and neurobehavioral symptoms measured using the Neurobehavioral Symptom Inventory (NSI).

Results: Baseline characteristics, including age (mean 47.4 ± 20.8 vs. 47.0 ± 21.8 years; p = 0.38), sex distribution (p = 0.14), and TBI severity (p = 0.15), were comparable between groups. Over six months, 11 patients (16.6%) in the BRV group and 17 patients (25.7%) in the LEV group experienced seizures (p = 0.20). Early PTS occurred in six BRV (9.1%) and 10 LEV (15.1%) patients (p = 0.32), while late PTS occurred in five BRV (7.5%) and seven LEV (10.6%) patients (p = 0.46). GOSE scores were similar at all follow-up points (overall mean 7.09 ± 1.78 vs. 7.06 ± 1.76; p = 0.93). However, BRV-treated patients showed significantly lower NSI scores at day 14 (10.74 ± 7.11 vs. 40.06 ± 4.93; p = 0.005) and three months (4.70 ± 4.78 vs. 13.23 ± 2.44; p < 0.001), with no significant difference at six months. The overall mean NSI score remained lower in the BRV group (5.72 ± 5.08 vs. 22.85 ± 2.89; p < 0.001).

Conclusions: BRV and LEV demonstrated comparable efficacy in preventing post-traumatic seizures in TBI patients. However, seizure incidence did not differ significantly in this sample. Although the study was underpowered for this outcome, BRV was associated with superior neurobehavioral outcomes, particularly during the early recovery period. These findings suggest that BRV may offer a more favorable tolerability profile for seizure prophylaxis following TBI. Larger multicenter studies are warranted to validate these results.

## Introduction

Traumatic brain injury (TBI) is a major global health concern, affecting approximately 69 million people annually, or nearly 8,000 individuals every hour [1-3]. It is a leading cause of mortality and long-term disability in both adults and children [4,5], most commonly resulting from motor vehicle accidents (50%), falls (21%), assaults and violence (12%), and sports or recreational activities (10%) [5]. TBI severity is classified according to the Glasgow Coma Scale (GCS), which assesses eye opening, verbal response, and motor function [5,6]. A score of 13-15 represents mild injury, 9-12 moderate injury, and 3-8 severe TBI [5]. Moderate-to-severe injuries carry a high mortality of 25-30%, and fewer than 50% of survivors regain full independent living [5,7]. Reflecting its long-term impact, the 2022 Lancet Commission proposed that moderate-to-severe TBI should be recognized as a chronic disease [5].

Post-traumatic seizures (PTSs) are a frequent complication of TBI and are classified based on onset: immediate (<24 hours), early (within seven days), or late (>seven days post-injury) [8]. The incidence of PTSs ranges from 4% to 14%, depending on injury severity, lesion location, and patient-specific risk factors such as age or prior epilepsy [9]. TBI is reported in over 40% of patients with functional seizures [10,11], and epilepsy risk increases 1.5-fold after mild TBI and up to 17-fold after severe injury [12]. Early post-traumatic seizures (EPTSs) are associated with prolonged intensive care and hospital stays and a greater likelihood of nursing home discharge [13,14]. Furthermore, EPTSs are a predictor of late PTSs and post-traumatic epilepsy, which may account for up to 20% of all epilepsy cases [15].

Current guidelines recommend antiseizure medication (ASM) prophylaxis for up to seven days following TBI [16] and evidence for reduction of late PTSs is limited. Some evidence suggests that starting ASMs within this period may also reduce late PTS incidence [17]. In the acute setting, agents must be available in both parenteral and oral formulations for immediate and continued use. However, uncertainty persists regarding optimal drug choice and duration of therapy. Phenytoin and levetiracetam (LEV) remain the most frequently prescribed agents, but LEV is associated with behavioral adverse events (BAEs) including irritability, aggression, and mood disturbances [18].

Brivaracetam (BRV), a newer ASM with high selectivity and strong affinity for the synaptic vesicle protein 2A (SV2A), has demonstrated efficacy in seizure control with potentially superior tolerability compared to LEV [19,20]. While LEV has favorable pharmacokinetics, adverse events, such as headache, fatigue, cognitive impairment, dizziness, insomnia, and behavioral changes, are common in TBI patients, in whom drug metabolism and brain vulnerability may differ from epilepsy populations [21]. BRV has been shown to reduce seizure frequency with fewer neurobehavioral side effects [22]. Importantly, switching from LEV to BRV has been associated with improvements in irritability, aggression, and psychiatric adverse events [23].

Following the recent availability of BRV in Pakistan, it is increasingly being used as monotherapy or adjunctive therapy for acute and long-term seizure management in epilepsy. This observational study aims to evaluate the clinical effectiveness of BRV in patients with TBI, specifically comparing its impact on seizure occurrence, seizure frequency, and drug-related adverse events against LEV when used for prophylaxis. The primary outcome is seizure incidence over six months, and secondary outcomes include neurobehavioral tolerability (NSI scores) and functional recovery (GOSE scores).

## Materials and methods

Study design and setting

This prospective cohort study was conducted in the Department of Neurosurgery at a tertiary care hospital. Approval from the institutional Ethics Review Committee (ERC) was obtained prior to study initiation.

Sample size calculation

The sample size was calculated using OpenEpi software (version 3.01). A minimum of 980 participants was required (490 in each group, with BRV as the exposed group and LEV as the non-exposed group), assuming a seizure incidence of 1% in the BRV group and 2.8% in the LEV group [24], with a significance level of 5% and 80% statistical power. However, due to the single-center setting and an approximate trauma admission rate of 110 patients every six months, the achievable recruitment was lower than initially projected. After applying a 20% inflation factor, a final sample size of 132 patients (66 per group) was obtained, so this study is underpowered for seizure incidence differences.

Participants

All patients presenting with suspected head trauma were managed according to Advanced Trauma Life Support (ATLS) 10th edition guidelines [25] and treated following the Brain Trauma Foundation, 4th edition recommendations [16]. Inclusion criteria were age greater than 18 years and a clinical diagnosis of TBI supported by neuroimaging evidence of acute intracranial trauma, including contusion, subdural, epidural, or intracerebral hemorrhage, or depressed skull fracture. Exclusion criteria comprised pre-existing epilepsy or prior seizures, history of neurosurgical procedures involving dural penetration, stroke, brain tumors, diabetic coma, or fatal injury. Pregnant and lactating women were also excluded. Eligible patients were enrolled through non-probability consecutive sampling after informed consent was obtained from the patient or their designated attendant.

Treatment groups

This prospective observational cohort study enrolled patients with TBI who met the eligibility criteria. Participants were assigned to treatment groups based on the antiepileptic drug (AED) selected by the treating physician; thus, no randomization or blinding was performed. Patients who received brivaracetam 50 mg twice daily were categorized as Group A (BRV group), while those receiving levetiracetam 500 mg twice daily comprised Group B (LEV group). Both medications were initiated intravenously at admission when feasible and transitioned to oral formulations upon clinical stabilization or discharge. Because treatment allocation was determined by physician preference and clinical judgment, the potential for selection bias exists, and the observed outcomes therefore represent associations rather than causal effects. Because treatment allocation was physician-driven, baseline factors known to influence seizures/NSI (CT lesion characteristics, presence of craniotomy or ICP monitoring, contusion burden, and timing of AED initiation) were carefully compared between groups and were largely balanced.

Outcome measures

The primary outcome was the occurrence of PTSs, defined as any seizure observed or documented by healthcare personnel after TBI. When present, seizures were classified as early (within seven days post-injury) or late (beyond seven days). Seizures were clinically noted during admission and after discharge were reported by patient or care givers. Secondary outcomes included functional status assessed using the Glasgow Outcome Scale-Extended (GOSE) and neurobehavioral symptoms measured using the Neurobehavioral Symptom Inventory (NSI), a patient-reported assessment tool for post-concussion symptoms [26,27]. GCS, GOSE and NSI are open-access instruments. TBI severity was categorized according to the Glasgow Coma Scale (GCS): mild (13-15), moderate (9-12), or severe (≤8).

Data collection procedures

Data were recorded using study-specific case report forms and included demographic information, clinical history, TBI severity, neuroimaging findings, seizure occurrence and frequency, GOSE scores, and NSI assessments. Patients were evaluated at four scheduled time points: baseline (Day 1), Day 14, three months, and six months. All assessments were performed during routine clinical visits; no additional visits were required for study purposes.

Statistical analysis

Data analysis was conducted using STATA version 15 (StataCorp LLC, College Station, Texas, USA). Quantitative variables such as age and NSI scores were summarized as mean ± standard deviation (SD) or median with interquartile range (IQR), depending on distribution normality assessed using the Shapiro-Wilk test. Categorical variables, including sex and seizure incidence, were described using frequencies and percentages. Group comparisons were performed using the chi-square test or Fisher’s exact test for categorical variables and Student’s t-test or the Mann-Whitney U test for continuous variables, as appropriate. A p-value of <0.05 was considered statistically significant.

## Results

A total of 132 patients were enrolled in the study, all of whom met the inclusion criteria and completed the six-month follow-up period. Patients who withdrew consent or did not complete follow-up were excluded. Participants were prospectively enrolled and assigned to two cohorts based on the antiseizure medication prescribed at the discretion of the treating physician: Group A (Brivaracetam, n=66) and Group B (Levetiracetam, n=66).

Group A comprised 13 female patients and 53 male patients, while Group B included seven female patients and 59 male patients, with no significant gender difference (p = 0.14). The age range of participants was 18 to 95 years, with a mean age of 47.42 ± 20.85 years in Group A and 47.03 ± 21.81 years in Group B (p = 0.38). Regarding injury severity, both groups had 45 patients with mild TBI. In Group A, 13 patients had moderate injuries and eight patients had severe injuries, whereas in Group B, 17 patients had moderate injuries and four patients had severe injuries. This difference was not statistically significant (p = 0.15) (Table 1). Radiologic injury patterns were similar between groups. In the BRV group vs LEV group, subdural hematoma was present in 28/66 (42.4%) vs 30/66 (45.5%), epidural hematoma in 9/66 (13.6%) vs 8/66 (12.1%), intracerebral hemorrhage in 14/66 (21.2%) vs 16/66 (24.2%), cerebral contusions in 22/66 (33.3%) vs 25/66 (37.9%), and depressed skull fracture in 6/66 (9.1%) vs 7/66 (10.6%), with no statistically significant differences between groups (all p > 0.05). Neurosurgical intervention was required in 18/66 (27.3%) patients in the BRV group and 20/66 (30.3%) in the LEV group (p = 0.70). These findings confirm comparable baseline injury phenotype and clinical course between cohorts.

**Table 1:** Baseline demographics and characteristics of both groups GOSE: Glasgow Outcome Scale-Extended

Variable	Group A (Brivaracetam) (n=66)	Group B (Levetiracetam) (n=66)	p-value
Gender			0.14 (χ² = 2.17)
Female n (%)	13 (19.7%)	7 (10.6%)	
Male n (%)	53 (80.3%)	59 (89.4%)	
Age, mean ± SD (years)	47.42 ± 20.85	47.03 ± 21.81	0.38 (t = 0.88)
TBI severity			0.15 (χ² = 3.76)
Mild n (%)	45 (68.2%)	45 (68.2%)	
Moderate n (%)	13 (19.7%)	17 (25.8%)	
Severe n (%)	8 (12.1%)	4 (6.0%)	
GOSE (14 days)	7.05 ± 1.76	6.98 ± 1.75	0.86 (t = 0.18)
GOSE (3 months)	7.11 ± 1.78	7.06 ± 1.77	0.92 (t = 0.10)
GOSE (6 months)	7.18 ± 1.74	7.09 ± 1.71	0.88(t = 0.15)
GOSE, mean ± SD	7.09 ± 1.78	7.06 ± 1.76	0.93(t = 0.09)

Functional recovery assessed using the Glasgow Outcome Scale-Extended (GOSE) was similar between groups at all follow-up points. At day 14, the mean GOSE scores were 7.05 ± 1.76 in Group A and 6.98 ± 1.75 in Group B (p = 0.86). At three months, scores were 7.11 ± 1.78 and 7.06 ± 1.76, respectively (p = 0.92). At six months, functional recovery further improved, with Group A showing 7.18 ± 1.74 and Group B 7.09 ± 1.71 (p = 0.88). The overall mean GOSE scores were 7.09 ± 1.78 for Group A and 7.06 ± 1.76 for Group B, again showing no significant difference (p = 0.93) (Table 1).

During the six-month observation period, 11 patients (16.6%) in the BRV group and 17 patients (25.7%) in the LEV group experienced one or more seizures (risk ratio (RR) of 0.65 and 95% CI 0.33-1.27). When classified by timing, early PTSs (within seven days of injury) were observed in six patients (9.1%) in the BRV group and 10 patients (15.1%) in the LEV group (RR of 0.60 and 95% CI 0.23-1.55), while late seizures (after seven days) occurred in five patients (7.5%) in the BRV group and seven patients (10.6%) in the LEV group (RR of 0.71 and 95% CI 0.23-2.18). The difference in both early and late seizure incidence between the two groups was not statistically significant (p = 0.32 and p = 0.46, respectively). Although seizures occurred more frequently among LRV-treated patients, this difference was not statistically significant (Fisher’s exact test p = 0.201; χ² test p = 0.287). Analysis of seizure frequency revealed that most patients had a single seizure episode. In the BRV group, eight patients experienced a single event and three had multiple episodes; in the LRV group, 15 had a single event and two had multiple episodes. Fisher’s exact test showed that this difference was statistically insignificant (p = 0.07) (Table 2).

**Table 2:** Seizure outcome comparison in both groups

Variable	Group A (Brivaracetam) (n=66)	Group B (Levetiracetam) (n=66)	p-value
Any seizure, n (%)	11 (16.7%)	17 (25.8%)	0.201 (χ = 1.63)
Seizure frequency			0.07(χ = 3.28)
Single episode	8 (72.7%)	15 (88.2%)	
Multiple episodes	3 (27.3%)	2 (11.8%)	

Neurobehavioral symptoms, assessed using the NSI, showed significant early differences between groups. At day 14, the mean NSI score was 10.74 ± 7.11 in the BRV group and 40.06 ± 4.93 in the LEV group (p = 0.005). At three months, scores improved in both groups but remained significantly lower in the BRV group (4.70 ± 4.78 vs. 13.23 ± 2.44; p < 0.001). At six months, both groups showed further improvement, with mean scores of 2.10 ± 2.65 in Group A and 3.01 ± 2.37 in Group B; this difference was not statistically significant (p = 0.19). The average NSI score across all follow-up points was 5.72 ± 5.08 in the BRV group and 22.85 ± 2.89 in the LEV group (p < 0.001), indicating better neurobehavioral outcomes with BRV (Table 3).

**Table 3:** Neurobehavioral Symptom Inventory (NSI) score comparison in both groups

Variable	Group A (Brivaracetam) (n=66)	Group B (Levetiracetam) (n=66)	p-value
NSI (14 days)	10.74 ± 7.11	40.06 ± 4.93	0.005 (t = 3.03)
NSI (3 months)	4.70 ± 4.78	13.23 ± 2.44	<0.001 (t = 4.85)
NSI (6 months)	2.10 ± 2.65	3.01 ± 2.37	0.19 (t = 1.33)
NSI, mean ± SD	5.72 ± 5.08	22.85 ± 2.89	<0.001 (t = 5.12)

## Discussion

TBI remains the leading cause of disability among all traumatic injuries [1]. Individuals with TBI are at increased risk of developing seizures, which have been linked to adverse outcomes such as hippocampal atrophy, coma, and encephalopathy [28]. The overall 10-year seizure risk following TBI has been reported as 4%, compared with 0.9% in controls [29]. This risk rises with injury severity, reaching 15-25% in patients requiring surgical decompression [30,31]. Although routine ASM prophylaxis after blunt TBI has not consistently demonstrated a reduction in seizure incidence [32], current guidelines recommend ASM use for seven days in severe TBI as PTS prophylaxis [16]. BAEs, including irritability, agitation, aggression, and emotional lability, are reported in 1-10% of patients receiving LEV [33].

Seizure prophylaxis aims to reduce early PTSs, limit disruptions in cerebral physiology, and potentially prevent long-term epilepsy, herniation, or mortality. However, the potential benefits must be balanced against the risk of neurobehavioral side effects, particularly if prophylaxis does not significantly alter seizure outcomes. Therefore, evaluating the comparative efficacy and tolerability of different ASMs is essential.

To date, only one prior study has directly compared BRV and LEV in TBI patients, focusing on early seizures [34]. Our study extends this by assessing seizure outcomes and tolerability over a six-month period. The mean participant age in our cohort (47 years) was similar to that reported by Pandya et al. (43 years) [34]. In our study, 16.6% of patients in the BRV group experienced seizures, comparable to the 16% reported by Pandya et al. The seizure incidence in the LEV group was 25.7%, also consistent with the 24% observed by Pandya et al. However, seizure frequency differed: in our cohort, most patients experienced multiple seizures, whereas Pandya et al. followed up patients for only seven days, which may explain the discrepancy. While Pandya et al. evaluated early PTSs limited to the first seven days after injury, our study extends follow-up to six months, thereby capturing both early and late PTSs as well as seizure recurrence beyond the acute hospitalization period. This longer observation window is clinically important because late seizures contribute substantially to long-term morbidity, treatment decisions, and patient quality of life and cannot be assessed within a seven-day framework. Additionally, prolonged follow-up allowed us to evaluate longitudinal tolerability and neurobehavioral outcomes, which are particularly relevant when antiseizure medications are continued beyond the acute phase. Thus, our data complement and extend the findings of Pandya et al. by providing insight into the sustained effectiveness and tolerability of BRV versus LEV across the subacute and chronic phases of TBI.

Functional outcomes were assessed using the GOSE. To our knowledge, no prior study has compared GOSE scores in TBI patients receiving BRV for seizure prophylaxis. Neurobehavioral side effects were evaluated using the NSI, a validated tool for post-concussion symptoms. Similar findings have been described in prior observational studies: Steinhoff et al. reported reduced irritability in patients switched from LEV to BRV [35], Hirsch et al. found a decrease in irritability/aggressiveness from 27% with LEV to 8% with BRV [36], and Theochari et al. observed resolution of psychiatric side effects in 77% of patients after switching [37]. Likewise, Steinhoff et al. reported improvement in BAEs in over half of patients transitioned to BRV [35].

The mechanisms underlying improved tolerability with BRV remain unclear. Unlike LEV, BRV lacks activity on neurotransmitter systems such as serotonin (5-HT) and does not act as an AMPA receptor antagonist, which may contribute to the lower incidence of BAEs [38]. Alternatively, differences in binding affinity or interaction with the SV2A protein may explain the observed clinical differences [35,39].

Our study is among the first to prospectively compare BRV and LEV for PTS prevention in TBI patients over a six-month follow-up, using both GOSE and NSI as outcome measures. These internationally recognized tools provide valuable insights into functional recovery and drug tolerability.

Although this study was designed to compare the efficacy and tolerability of BRV and LEV for PTS prophylaxis, the sample size achieved (n=132) was smaller than the originally calculated requirement of 980 participants. As a result, the study is underpowered to detect small differences in seizure incidence, and the lack of statistical significance for the primary outcome should be interpreted cautiously. Also prescribing patterns may have been influenced by local availability and clinician preference, which may limit external applicability. Additionally, because the majority of enrolled patients had mild TBI, our findings are most directly generalizable to mild TBI populations, and caution is warranted when extrapolating these results to patients with moderate or severe injuries. Also as a non-randomized, physician-discretion cohort, selection bias is possible, as treatment choice may have been influenced by perceived seizure or behavioral risk. Lack of blinding may also affect subjective outcomes, limiting causal inference.

However, this study provides valuable preliminary data supporting the comparable efficacy of BRV and LEV, with a notable advantage of BRV in neurobehavioral tolerability. These findings establish a foundation for further research and have informed the design of a larger, prospective, multicenter follow-up study that is currently being planned. This upcoming study aims to achieve adequate statistical power to more definitively assess differences in seizure prevention and behavioral outcomes following TBI

## Conclusions

In this study, BRV and LEV demonstrated generally comparable seizure outcomes following TBI. While seizure incidence and frequency were not statistically different between groups, BRV was associated with significantly better neurobehavioral outcomes, as reflected by lower NSI scores. These findings suggest that BRV may provide a more favorable safety and tolerability profile compared to LEV, particularly in minimizing BAEs. Larger, multicenter randomized controlled trials are warranted to validate these results and further establish the role of BRV in seizure prophylaxis after TBI as the conclusions about seizure prevention are not definitive due to underpowering and non-randomization.
